# Mutations in Tau’s Proline Rich Domain Affect its Protein Interactions

**DOI:** 10.1002/alz.089653

**Published:** 2025-01-03

**Authors:** Anne Koutures, Brittany Katz, Kaylin Hwang, Whitaker Cohn, Kwaku Addo‐Osafo, Keith Vossel

**Affiliations:** ^1^ UCLA, Los Angeles, CA USA; ^2^ University of California, Los Angeles, Los Angeles, CA USA; ^3^ Drug Discovery Lab, Mary S. Easton Center for Alzheimer’s Disease Research at UCLA, Los Angeles, CA USA; ^4^ University of California, Los Angeles (UCLA), Los Angeles, CA USA; ^5^ University of California, Los Angeles, CA USA; ^6^ University of California Los Angeles, Los Angeles, CA USA

## Abstract

**Background:**

Epileptic activity is increasingly recognized as a contributor to Alzheimer’s Disease (AD) pathology. In AD models, endogenous tau contributes to epileptic activity and associated cognitive deficits through mechanisms that are not fully understood. Increased attention is being directed towards tau’s interactions with proteins that regulate neuronal activity, particularly tau’s proline rich domain and its binding to SH3‐containing proteins. However, the impact of mutations in tau’s proline‐rich region on other protein interactions remains unclear.

**Method:**

We assessed mutations in tau’s proline rich domain in two mouse lines: AxxA6 (P‐to‐A substitutions in 6th PxxP motif in tau) and R221A (R‐to‐A substitution at amino acid corresponding to 221 in human 2N4R tau). These mutations block tau’s interactions with SH3‐containing proteins, including PLCγ1 and p85α/PI3K. Co‐immunoprecipitation (Co‐IP) of tau from cortical brain tissue was confirmed and analyzed by mass spectrometry. We compared protein abundance ratios to wild type tau mice within each mouse line. Significant (p<.05) tau‐baited protein interactions were further explored within literature and the String Database.

**Result:**

The R221A line produced 143 and 120 significant proteins within the heterozygous and homozygous groups, respectively. Many identified proteins are strongly associated with processes such as microtubule organization, postsynaptic structure, and mitochondrial functions. The AxxA6 line produced 158 and 126 significant proteins within the heterozygous and homozygous groups, respectively. Many identified proteins are involved in cellular trafficking and are strongly associated with processes such as glutamate catabolic processes and microtubule protein localization. Notably, many proteins identified in both lines are involved in prion disease and vascular endothelial growth factor (VEGF) signaling pathways.

**Conclusion:**

Co‐IP mass spectrometry of the R221A and AxxA6 mouse lines revealed that mutations in tau’s proline rich domain to affect tau’s interactions with multiple proteins. Of the significant proteins, multiple are related to microtubule, metabolic, synaptic, and mitochondrial activity, and many are found within prion disease pathways and VEGF signaling pathways. Interestingly, PLCγ1 and p85α/PI3K, are also implicated in VEGF signaling pathways. These findings highlight the importance of further investigating the possible relationships between noted cellular processes and biological pathways and tau‐protein interactions in familial AD.

